# Effect of Cutting Conditions on the Size of Dust Particles Generated during Milling of Carbon Fibre-Reinforced Composite Materials

**DOI:** 10.3390/polym16182559

**Published:** 2024-09-10

**Authors:** Štěpánka Dvořáčková, Dora Kroisová, Tomáš Knápek, Martin Váňa

**Affiliations:** Assembly and Engineering Metrology, Department of Machining, Faculty of Mechanical Engineering, Technical University of Liberec, 461 17 Liberec, Czech Republic

**Keywords:** CFRP, milling, electron microscopy, dust particles, respiratory hazards

## Abstract

Conventional dry machining (without process media) of carbon fibre composite materials (CFRP) produces tiny chips/dust particles that float in the air and cause health hazards to the machining operator. The present study investigates the effect of cutting conditions (cutting speed, feed per tooth and depth of cut) during CFRP milling on the size, shape and amount of harmful dust particles. For the present study, one type of cutting tool (CVD diamond-coated carbide) was used directly for machining CFRP. The analysis of harmful dust particles was carried out on a Tescan Mira 3 (Tescan, Brno, Czech Republic) scanning electron microscope and a Keyence VK-X 1000 (Keyence, Itasca, IL, USA) confocal microscope. The results show that with the combination of higher feed per tooth (mm) and lower cutting speed, for specific CFRP materials, the size and shape of harmful dust particles is reduced. Particles ranging in size from 2.2 to 99 μm were deposited on the filters. Smaller particles were retained on the tool body (1.7 to 40 μm). Similar particle sizes were deposited on the machine and in the work area.

## 1. Introduction

Composite systems are used in many industrial areas, from stall building to construction. Thanks to the mechanical parameters mediated by, for example, high quality carbon, glass, basalt, aramid and natural fibres embedded in reactive, thermoplastic, geopolymer and cement matrices, it is possible to achieve improved mechanical parameters, weight reduction, increased resistance to dynamic stresses and more. Despite efforts to design and manufacture so-called “made-to-order” components, many manufactured components have to be modified by so-called finishing operations—milling, drilling or grinding [1].

The amount of dust generated during dry machining of CFRP depends on the nature of the machining process, cutting speed, feed rate, geometry and material of the cutting tool, workpiece and orientation of the workpiece grain. Dry machining of CFRP causes not only more intensive wear of the cutting tool but also, and above all, an increase in the emission of fine dust particles from the machined material (chips) with extremely small dimensions and sharp shapes entering the air [2]. In terms of the machining operations performed, drilling produces lower concentrations of dust particles than milling but higher concentrations than grinding. Dust generated during composite machining can negatively affect human health. Skin exposure, inhalation, ingestion of broken fibres and fine particles can lead to health complications. Several toxicological studies carried out on rabbits and rats confirm that composite dust has adverse effects on the lungs [3,4,5].

**Figure 1:**
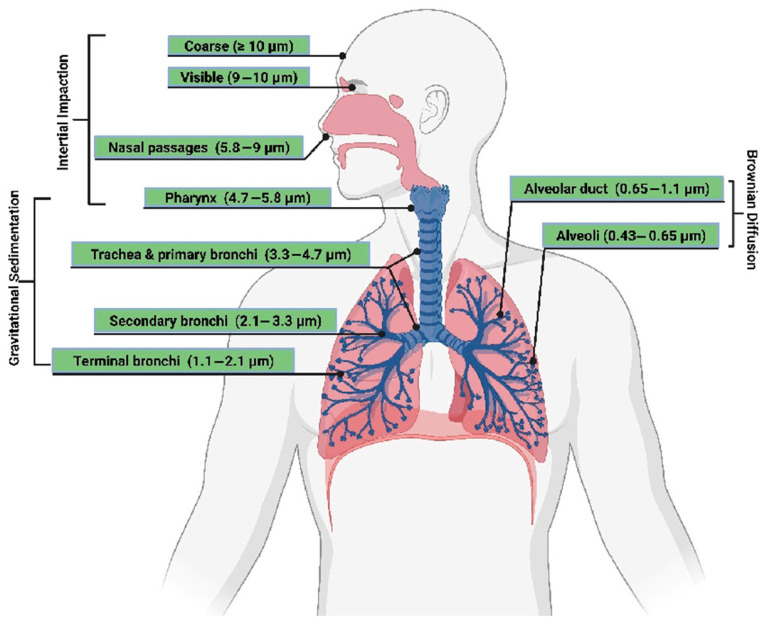
Size of inhaled particles. Reproduced from [9], MDPI, 2023.

Generally, a particle is considered to be non-breathable as opposed to unbreathable if its aerodynamic diameter is greater than 10 µm. However, several studies have distinguished the respirable nature of a particle according to where the particle settles in the airways, as in Figure 1. Almost all particles larger than 10 µm are trapped in the nasal cavity [6]. Most particles with a size between 5 and 10 µm settle in the nasopharynx or larynx. Approximately 40% of particles with a particle size between 2 and 5 µm settle between the trachea and bronchi. Particles smaller than 2 µm have a high probability of reaching the bronchi and lung chambers from which they can then enter the bloodstream. The particles with the greatest potential to damage the lungs are those in the lung chamber area because they are very close to the lung wall [7,8].

Cutting conditions in milling CFRP materials such as cutting speed, feed per tooth and depth of cut affect the size, shape and amount of fine dust particles [10]. These airborne dust particles are considered carcinogenic and have the potential to damage the respiratory system and cause toxicity and irritation. Therefore, it is very important to identify the main technological factors responsible for the generation of harmful dust particles so as to minimize their emission into the environment during industrial CFRP machining. In the machining of heterogeneous CFRP, studies [4,11,12,13] have reported that the presence of dust is less than the theoretical chip thickness. The size, shape and amount of dust particles increase or decrease with increasing or decreasing cutting speed and with increasing or decreasing feed rate or feed per tooth when machining these materials. Different studies give different recommendations, but it is good to keep in mind that each study is for a different type of material to be machined (weave, number of layers, fabric saturation with epoxy resin, fabrication, etc.) with different cutting tools (geometry, material, etc.). Of course, if we apply high-speed machining, dust increases 2–8 times. On the other hand, a higher feed rate generates less dust due to rough cutting [14,15]. The above studies show that dust particles that are smaller than 10 μm in size and shape are dangerous for the operator of the machining operation.

The use of protective clothing, respirators and protective masks is essential when machining composite materials with carbon and glass fibre reinforcement. The handling of protective equipment contaminated with larger or smaller particles of reinforcing fibres is, consequently, a problem that needs to be considered. The fibres are easily released and can subsequently enter the surrounding environment. Sub-micrometre particles that are shed from the ends of the fibres can be a significant problem and can pass through inadequately selected filtration systems or inadequate protective work equipment. The collection of these destroyed fibre systems is important from a health perspective. The subsequent usefulness of the fibres thus recovered is questionable as they are unlikely to have the required slenderness ratio to be used as short fibres in polymer matrices and the cost of recovering them from filters is likely to exceed a reasonable limit.

Machining processes are commonly carried out in enclosed machines that significantly reduce the release of hazardous dust. Broken fibres of all sizes enter the process liquids, which are taken to collection yards and subsequently disposed of. The risk is reduced but not eliminated with liquid disposal.

When evaluating particle formation due to destruction during machining, the influence of the system matrix and the fibres used and their distribution is evident. Reactoplastic matrices are easily crushed, whereas thermoplastic matrices melt and coat the broken fibres, increasing their size and reducing the potential hazard to humans.

The present study deals with the combination of different cutting speeds and feeds per tooth using constant radial depth of cut and their effect on the size, shape and quantity of hazardous dust particles generated during side milling of CFRP.

This study did not focus on the spatial distribution of dust particles, i.e., the density of dust in the space of a room or building.

## 2. Materials and Methods

### 2.1. Composite Material

**Figure 2:**
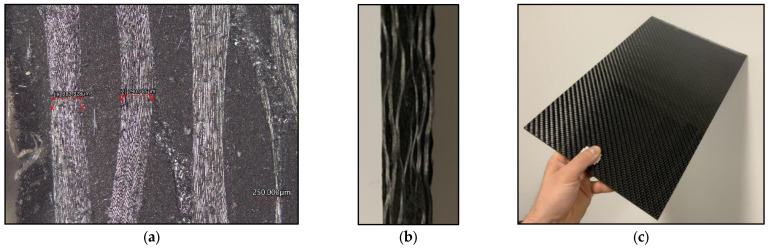
Machined composite material: (**a**) 250 μm composite plate cut; (**b**) plate cut; (**c**) composite plate.

The composite material used for this experiment was a carbon fibre-reinforced polymer, see Figure 2. The CFRP laminate was made using unidirectional prepregs—see Table 1. The laminate was made by vacuum infusion, where the base was a CCh600 (Kordcarbon, Strážnice, Czech Republic) type reinforcement (carbon fibre) and a LG120 (GRM Systems, Olomouc, Czech Republic) type matrix as an epoxy resin. This is the material used to manufacture some primary and secondary aircraft parts.

The laminates were cut into test specimens of 300 mm × 150 mm × 4.5 mm (length × width × specimen thickness). These parameters do not affect the dust particles generated during machining.

### 2.2. Cutting Tool, Cutting Parameters

**Figure 3:**
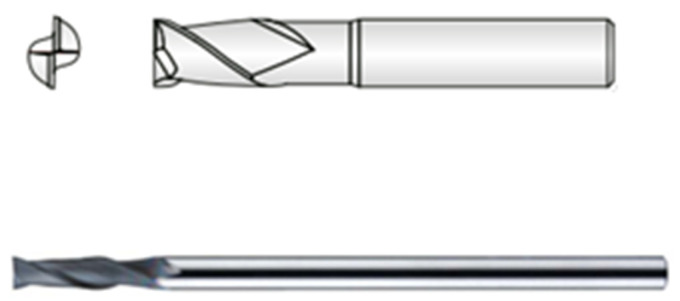
Cutting tool.

In order to investigate the effects of cutting conditions on the size, shape and amount of particles released, a suitable cutting tool was first selected. The cutting tool was a 2-edged 6 mm diameter carbide shank cutter with CVD diamond coating. The specific tool information is given in Table 2 and Figure 3. This cutting tool was selected as the most used and suitable for machining carbon fibre polymer based composite materials.

In order to study the effect of cutting parameters on the harmful dust generated, three levels of cutting speed (120, 170 and 220 m/min) and three levels of feed per tooth (0.05, 0.1 and 0.2 mm) were selected. A radial depth of cut of 1 mm was used for all these cutting speeds and feeds per tooth.

In order to simulate a real industrial process, lubricant-free machining (dry machining) and a side-by-side milling type was implemented. Contiguous milling was carried out at an angle of 0°. The details of the experimental cutting parameters are given in Table 3.

For each cutting condition, three samples were prepared, giving a total machining path of 1800 mm (2 × 300 mm × 3 samples = 1800 mm). For each sample, constant cutting conditions were maintained, including the cutting direction.

### 2.3. Machining Machine

The milling process was carried out on a 3-axis CNC milling machine from the manufacturer DMG MORI CMX 600V (DMG Mori Seiki, Nagoya, Japan) with a maximum spindle speed capacity of 12,000 rpm. The specimens were securely mounted using head screws in a fabricated jig commonly used for milling composite materials.

#### Exhaust Equipment

Dust generated during machining was extracted using a device designated POC9 M1 (Vzduchotechnik, Chrastava, Czech Republic), the parameters of which are given in Table 4.

### 2.4. Collection and Analysis of Dust Particles

Harmful dust particles generated during the milling process were collected from the filter of the extraction system, from the cutting tool and from the workpiece and jig.

A special non-woven filter for the experiment was used to capture the dust particles, the parameters of which are given in Table 5. 

After each machining test, the generated harmful dust particles were analysed using a SEM and a Keyence VK-X 1000 (Keyence, Itasca, IL, USA) confocal microscope. The Keyence VK-X 1000 confocal microscope was used to obtain 3D images of the different sizes and shapes of the dust particles and also to investigate cutting tool wear (geometric measurements of the cutting edges).

Prior to the milling process, the workpiece samples were carefully oriented with respect to cutting speed and feed rate per tooth compared to the orientation of the layers. Thus, the angle between the layers that form the laminate are kept constant compared to the direction of the cutting speed.

## 3. Results

According to European standard EN 481 [16], dust particles are divided into 3 categories: inhalable, thoracic and respirable, where respirable dust are those particles that can reach the lung and alveoli and are characterized by aerodynamic diameters less than 10 µm. This category of dust particles poses a serious health risk to operators carrying out machining operations. For this reason, it is very important to address this issue and to try to reduce the amount of dust particles released during machining operations.

In this study, the harmful dust particles generated by CVD diamond coated carbide tools at the cutting conditions given in Table 3 are analysed in terms of size, shape and quantity.

### 3.1. Effect of Cutting Conditions on the Size of Dust Particles on the Filter of the Extraction Device

Harmful dust particles generated during the milling process were collected and analysed using a SEM and a Keyence VK-X 1000 confocal microscope after each milling test.

After milling, particles on the filter of the extraction device, on the cutting tool and on the workpiece and jig were analysed. The purpose of this study was not to analyse harmful dust particles suspended in the air.

#### 3.1.1. Effect of Cutting Conditions on the Size of Dust Particles on the Exhaust Filter

**Figure 4:**
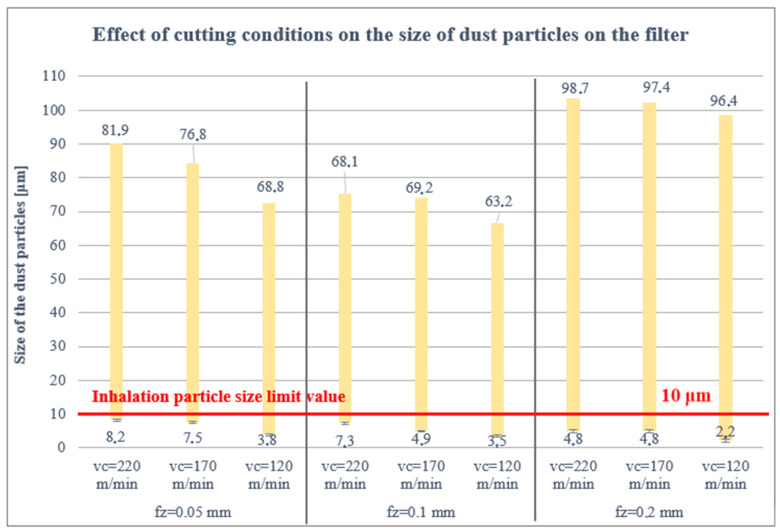
Size of particles trapped on the filter.

The real measured sizes of the collected dust particles on the filter for the cutting conditions used with the CVD diamond coated carbide tool are presented in Figure 4. Figure 4 shows that the smallest harmful dust particles, below 8 μm, were measured in the extensive particle analysis on the filter at the lowest cutting speed of 120 m/min and the highest feed per tooth of 0.2 mm. Conversely, the lower the feed per tooth, i.e., fz = 0.05 mm, and the higher the cutting speed, vc = 220 m/min. the larger the dust particle size, i.e., 8.2 μm. 

The increase in particle size in the case of fz = 0.2 compared to fz = 0.05 and fz = 0.1 is probably because at higher feed per tooth under the given conditions, parts of the material system of a larger size are cut off; these are more stable and the fragments stick together more. This identifies particles larger than in the fz = 0.05 and fz = 0.1 cases.

The collected dust particles on the filter were also analysed for their shape and selected parameters on a SEM. The analysis revealed that the adhesion between the polymer matrix and the carbon fibres was low, which may have been due to insufficient fibre saturation during manufacturing. It was also found that the fibres were grafted transversely or at an angle of 40–60°, i.e., with a pointed end [4]. The fibres had a diameter of 7 μm. The smallest particles were produced by chipping off pieces at the ends of the fibres. No significant effect of the different cutting conditions on fibre cleavage was observed. 

**Figure 5:**
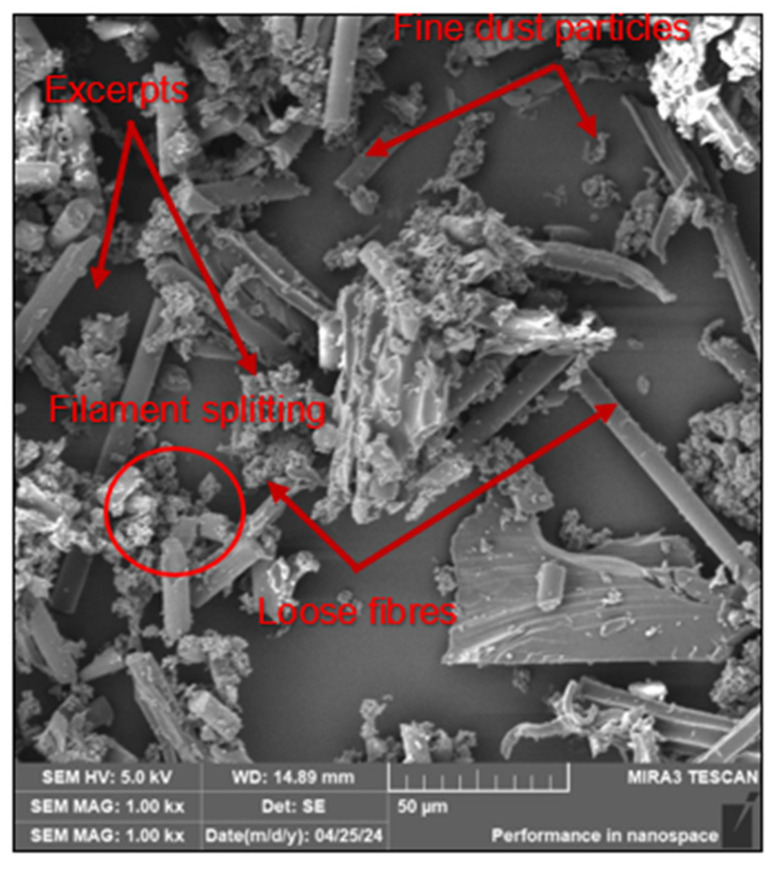
The SEM analysis of dust particles trapped on the filter for selected cutting conditions.

The analysis confirmed the occurrence of 3 types of dust particles: clusters of fibres denoted here as fragments, fine dust particles generated by chipping from fibre ends and free fibres of different sizes, see Figure 5.

#### 3.1.2. Effect of Cutting Conditions on the Size of Dust Particles on the Cutting Tool

**Figure 6:**
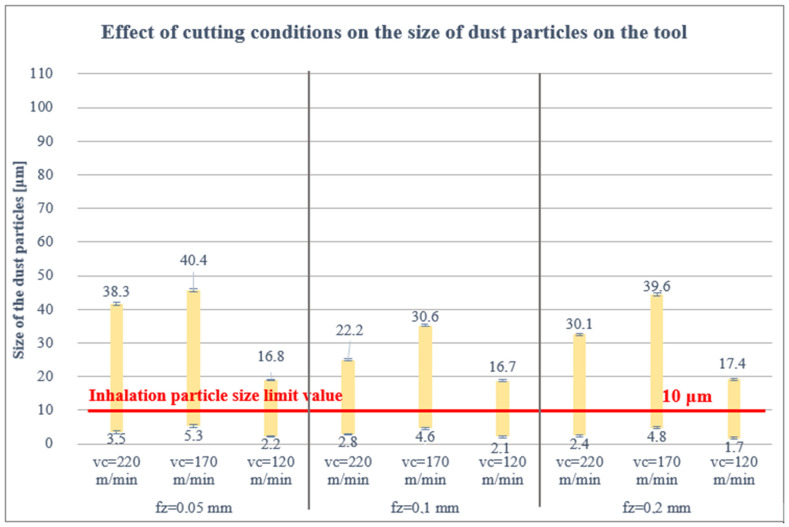
Particle size trapped on the cutting tool.

Much smaller dust particles were measured on the cutting tool than on the filter, Figure 6. This can be explained by the fact that the extraction device sucked out a significant part of the dust particles, even those with a larger shape and thus a larger mass, which do not tend to stick under their own weight to the tool. Figure 6 shows that the smallest harmful dust particles in the analysis performed, less than 5 μm, were again measured at the lowest cutting speed of 120 m/min and the highest feed per tooth of 0.2 mm, i.e., 1.7 μm. Conversely, the lower the feed per tooth, i.e., fz = 0.05 mm and the higher the cutting speed, vc = 170 m/min, the larger the size of the dust particles, i.e., 5.3 μm.

The SEM analysis in terms of the shape and overall properties of the dust particles on the cutting tool confirmed that the adhesion between the polymer matrix and the carbon fibres is low. Furthermore, the fibres were split transversely rather than at an angle of 40–60°. The particles were found to have more pieces with the fibre matrix than those trapped on the filter of the extraction device. This can probably be explained by the fact that during the milling process the tool and workpiece were heated and therefore the matrix adhering with the fibres was destroyed.

**Figure 7:**
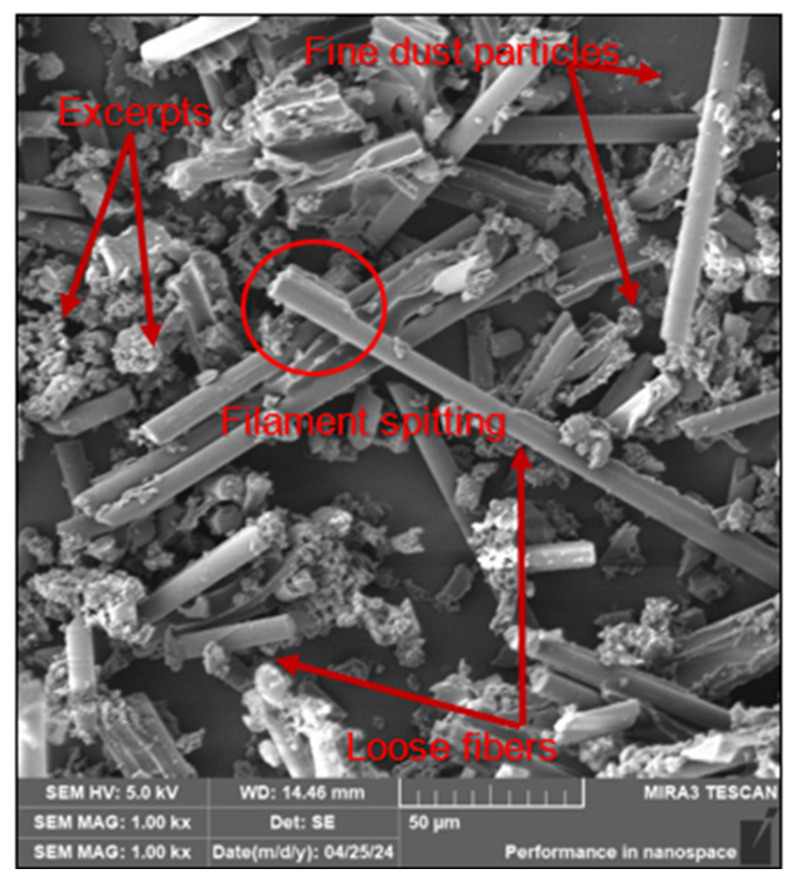
The SEM analysis—dust particles trapped on the cutting tool for selected cutting conditions.

The analysis confirmed again the occurrence of 3 types of dust particles with respect to the chosen cutting conditions, fragments, fine dust particles and free fibres of different sizes, see Figure 7.

#### 3.1.3. Effect of Cutting Conditions on the Size of Dust Particles on the Workpiece and Workpiece

**Figure 8:**
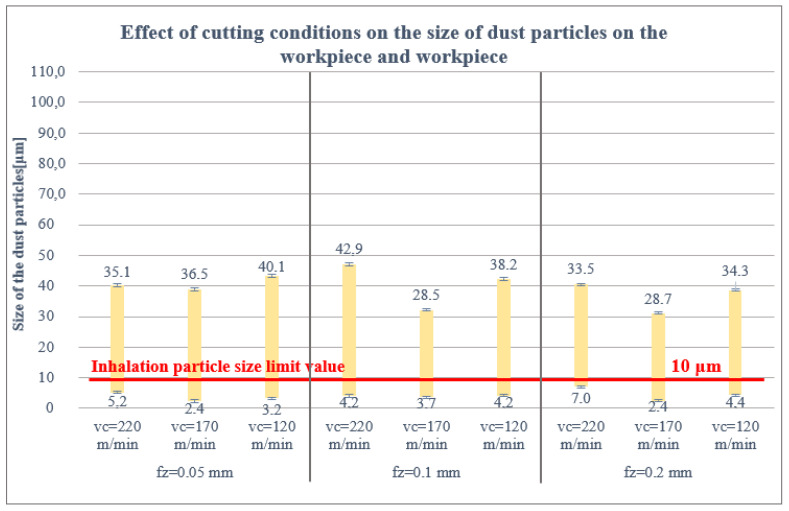
Size of particles trapped on the workpiece and fixture.

The smallest dust particle size trapped on the workpiece and jig was again measured at the highest feed per tooth of 0.2 mm and at a lower cutting speed of 170 m/min, i.e., 2.4 μm, Figure 8.

The SEM analysis of the collected dust particles on the workpiece and jig showed identical results to the filter and cutting tool. The fibres were split transversely or at an angle of 40–60°. No significant effect of the cutting conditions on the cleavage of fibres trapped on the cutting tool was observed. The analysis also confirmed the presence of 3 types of dust particles as on the filter and cutting tool. These particle types were identical for all cutting conditions studied.

In the extensive measurements, it was found that the size and shape of the harmful dust particles were more or less identical on the filter, cutting tool, workpiece and jig under the given cutting conditions. For the cutting tool, the workpiece and the jig, the particle sizes were evenly distributed (from 1.7 to 43 μm). 

Thus, for the next experiment, the study focused only on the collection of harmful dust particles generated during the milling process from the filter of the extraction device.

### 3.2. Effect of Cutting Tool Wear at Selected Cutting Conditions (vc =120 m/min and fz = 0.2 mm) on Dust Particle Size

Dust particles were collected only from the filter under cutting conditions and analysed using a SEM and a Keyence VK-X 1000 confocal microscope after each milling test, i.e., along 0.25, 0.5, 0.75, 1, 1.25 and 1.5 m paths.

**Figure 9:**
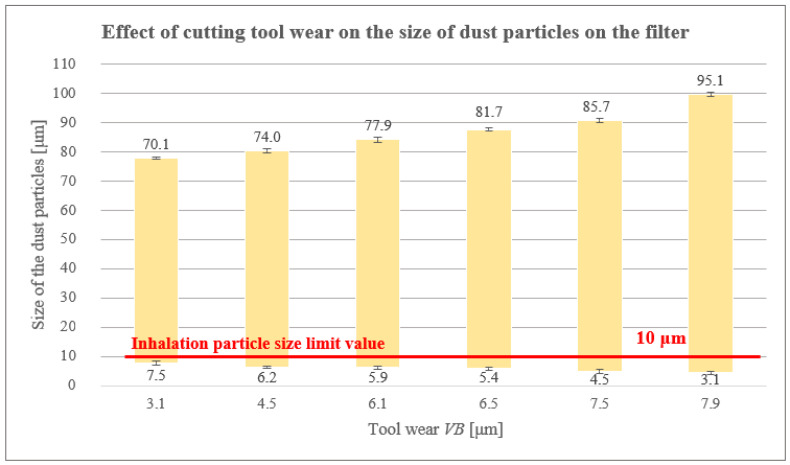
Size of particles trapped on the filter of a worn cutting tool.

From the measurements carried out (Table 6 and Figure 9), it is clear that the size of harmful dust particles decreased with increasing cutting path and cutting tool wear. The wear of the cutting tool was in the form of abrasion, caused by abrasive carbon fibres [17]. There were always small grooves and localized potholes on the cutting tool surface, caused by the mechanism of the carbon fibre bending and buckling away from the cutting point. The smallest harmful dust particles of 3.3 μm were measured on the filter at the longest cutting path of 1.5 m with a tool wear of 7.9 μm.

As the cutting path increased, the radius of the cutting edge at the cutting tool increased and thus the tool wear VB [μm] increased.

**Figure 10:**
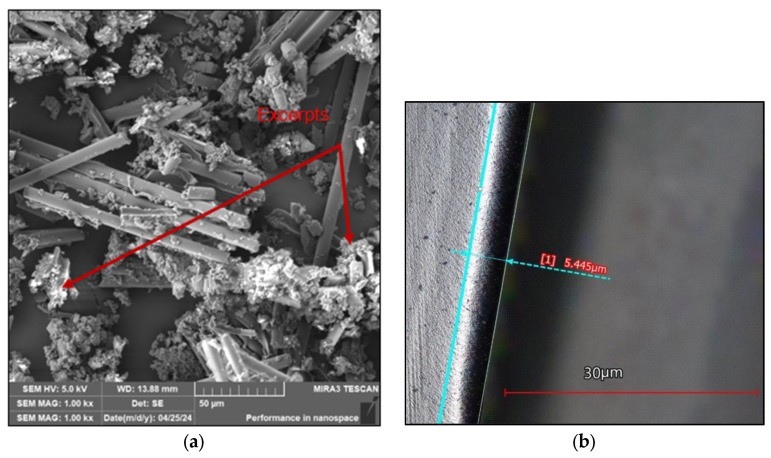
The SEM analysis: (**a**) dust particles trapped on the filter of the worn cutting tool; (**b**) worn cutting tool.

The generated dust particles were largely in the form of fragments (from fibres and matrix), Figure 10.

## 4. Discussion

### 4.1. Cutting Conditions

As already mentioned, a suitable combination of cutting conditions, i.e., cutting speed, feed per tooth and depth of cut, is necessary to reduce the size, shape and number of harmful particles. However, it is important to note that there is insufficient evidence in the literature on the effect of depth of cut on the resulting dust particle levels. However, research by Nguyen-Dinh, N. et al. [3] reported that when the radial depth of cut ranges from 1 mm to 3 mm there is a decrease in dust particle counts with increasing depth of cut. The explanation is that with higher value of depth of cut (e.g., greater than 1, e.g., 3), larger fragments of fibrous matrix are produced which have higher mass. Due to their higher mass, they cannot disperse in the air and settle on the machine table. The higher chip thickness therefore results in a smaller proportion of dust particles dispersed in the air.

### 4.2. Amount of Dust Particles Analysis

The whole study was very challenging in terms of the analysis of the amount of dust particles, i.e., detailed analysis/measurements of dozens of dust particles and their frequency on the filter, cutting tool, workpiece and jig.

The results of the study showed that the highest number of particles was captured on the filter, as expected. 

The proportion of dust particles captured on the filter was analysed as follows: 85% of the dust particles were larger than 10 μm, 13% of the dust particles were 5–10 μm and 2% of the dust particles were smaller than 5 μm. For the cutting tool, 41% of the dust particles were greater than 10 μm, 36% of the dust particles were 5–10 μm, 23% of the dust particles were less than 5 μm and the same for the workpiece and jig.

Analysis of the dust particles on the filter, cutting tool, workpiece and jig confirmed the presence of 3 types of dust particles: fibre clusters or fragments, fine dust particles generated by chipping off the ends of fibres that have been coated with matrix, and loose fibres of different sizes.

### 4.3. The Limit Value for Dust Particle Inhalation

As mentioned above, the limit value for dust particle inhalation is 10 μm, so attention was paid to the smallest dust particle sizes at the proposed cutting conditions (vc = 120, 170, 220 m/min; fz = 0.05, 0.1, 0.2 mm; ae = 1 mm). The results obtained show that the size and health damaging particles decrease up to 1.7 μm with increasing the feed rate per tooth (from 0.05 to 0.2 mm) and decreasing the cutting speed (from 220 to 120 m/min). This means that the combination of low cutting speed and high feed rate per tooth produces small, tiny and very dangerous harmful dust particles (those between 5 and 10 µm that can settle in the nasopharynx or larynx, between 2 and 5 µm that can settle between the trachea and bronchi and those particles smaller than 2 µm that can enter the bloodstream). However, it should be pointed out that the combination of these parameters causes mechanical damage (matrix, fibres) which, in turn, causes significantly poor surface quality. Of course, the geometry, material and coating of the tool also have an influence, promoting the adhesion of carbon and matrix dust on the tool. 

SEM analysis confirmed 3 types of dust particles: namely, fibre clusters or fragments, fine dust particles generated by chipping off the ends of fibres that have been coated with matrix and loose fibres of different sizes.

It was further found that the size of the harmful dust particles was more or less identical under the given cutting conditions on the filter, cutting tool, workpiece and jig. For the cutting tool, the workpiece and the jig, the particle sizes were uniformly distributed (from 1.7 to 43 μm). However, all measured harmful particles lie below 10 μm.

For the next experiment, the study focused only on the collection of harmful dust particles generated during the milling process from the filter of the extraction device.

In the measurements performed, it was shown that the size of the harmful dust particles decreased with increasing cutting path and cutting tool wear. As the cutting path increased, the cutting edge radius of the cutting tool increased and, thus, tool wear increased. The value of the cutting edge radius increased and became higher compared to the diameter of the carbon fibre (7 µm). This was associated with an increase in the value of cutting forces. As a consequence, the chip formation mechanism changed, with fibre bending and buckling, in contrast to the shear mechanisms observed with the new tool (with a 3 µm cutting edge radius). As the cutting edge radius increased, dust particles were produced in the form of loose fibres and larger pieces of matrix with fibres.

Based on these results, we can highlight the strong link that exists between cutting tool wear and the size and shape of harmful dust particles generated during milling. The dust particles generated were largely in the form of fragments.

## 5. Conclusions

An experimental study on the effect of cutting conditions (cutting speed, feed per tooth, depth of cut) and cutting tool wear on the size, shape and amount of harmful dust particles during milling of specific CFRP materials provides new insights into the machining of CFRP materials.

A CVD diamond coated carbide cutting tool was used to analyse the dust in terms of size, shape and amount released during milling of CFRP composite. 

The following conclusions can be drawn from the study:

- The SEM imaging analysis performed on the captured dust particles released during milling revealed the presence of 3 forms of dust particles/chips, viz. fibre clusters/fragments, fine dust particles generated by chipping off the ends of the fibres and loose fibres of various sizes. Furthermore, the analysis showed that the carbon fibres split transversely and at an angle with an inclination of 40–60° at a ratio of 50:50.
- Increasing the feed per tooth from 0.05 to 0.2 mm and decreasing the cutting speed from 220 to 120 m/min promotes a reduction in the size and shape of dust particles, i.e., the formation of small, tiny and very dangerous dust particles that can reach the operator’s bloodstream.
- The shape and size of the dust particles are significantly influenced by the geometry and coating of the cutting tool. A larger cutting edge radius (e.g., 6 µm) generates coarser dust particles (larger size) which, due to their higher mass, fall on the machine table and do not escape into the air or the operator’s respiratory system.
- The size of harmful dust particles decreased with increasing cutting path and increasing wear of the cutting tool. The cutting tool wear was in the form of abrasion caused by abrasive carbon fibres.

Overall, cutting conditions have been shown to have a significant effect on the size, shape and quantity of dust particles. It has also been shown that, regardless of where the dust particle collection is carried out, the shape and size of the dust particles follows the trend of the cutting conditions, i.e., small, tiny and very hazardous dust particles are formed as the feed rate per tooth increases and the cutting speed decreases.

Based on the above results, it can be concluded that milling carbon fibre composite materials with a feed rate per tooth of 0.2 mm and a cutting speed of 120 m/min creates hazardous health conditions for operators, as in Table and Figure 1.

## Figures and Tables

**Table 1:** Parameters of machined composite material.

Parameters	Value
Thickness [mm]	4.5
Width [mm]	42
Length [mm]	250
Production method	Vacuum infusion
Matrix	Epoxy resin LG120
Hardener	HG 356
Reinforcement	CCH600
Reinforcement grammage [g/cm^2^]	600
Type of weave	Twill 2/2

**Table 2:** Cutting tool parameters.

Parameters	Value
Tool diameter [mm]	6
Cutting length [mm]	18
Length [mm]	60
Helix angle [°]	30
Coating	CVD

**Table 3:** Cutting conditions.

Parameters	Value
Cutting speed [m/min]	120, 170, 220
Feed per tooth [mm]	0.05, 0.1, 0.2
Radial cutting depth [mm]	1

**Table 4:** Parameters of the extraction device POC9 M1.

Parameters	Value
Suction pressure [Pa]	900
Extracted air quantity [m^3^/h]	1200
Electric motor power [kW]	0.7
Noise level [dB]	65
Minimum particle capture size [µm]	0.3

**Table 5:** Nonwoven fabric parameters.

PEGATEX—PFNonwovens (Nonwoven Fabric)	
Material	Pegatex S anitsat
Material number	408243
Batch number	TRZ0A13747
Weight [gsm]	17

**Table 6:** Dust particle size for a worn cutting tool.

Path [m]	VB [μm]	Smallest Particle Size [μm] ± Measurement Uncertainty	Largest Particle Size [μm] ± Measurement Uncertainty
0.25	3.1	7.5 ± 0.9	70.1 ± 0.4
0.5	4.5	6.2 ± 0.4	74.0 ± 0.6
0.75	6.1	5.9 ± 0.7	77.9 ± 0.9
1	6.5	5.4 ± 0.4	81.7 ± 0.6
1.25	7.5	4.5 ± 0.6	85.7 ± 0.8
1.5	7.9	3.1 ± 0.7	95.1 ± 0.7

## Data Availability

The original contributions presented in the study are included in the article, further inquiries can be directed to the corresponding author/s.
